# Supplementary material for: Optofluidic crystallithography for directed growth of single-crystalline halide perovskites
Source: Nat Commun. 2024 May 1;15:3677. doi: 10.1038/s41467-024-48110-w (PMC11063063; doi:10.1038/s41467-024-48110-w)
Supplement: Supplementary file 1 — Supplementary Information [file 41467_2024_48110_MOESM1_ESM.pdf]

Supplementary Information

Optofluidic crystallithography for directed growth of single-crystalline halide perovskites

Xue-Guang Chen[1,2], Linhan Lin[2]*, Guan-Yao Huang[3], Xiao-Mei Chen[2], Xiao-Ze Li[2], Yun-Ke Zhou[2], Yixuan Zou[2], Tairan Fu[3], Peng Li[2], Zhengcao Li[1]*, and Hong-Bo Sun[2,4]*

[1] Key Laboratory of Advanced Materials (MOE), School of Materials Science and Engineering, Tsinghua University, Haidian, Beijing, 100084, China

[2] State Key Laboratory of Precision Measurement Technology and Instruments, Department of Precision Instrument, Tsinghua University, Haidian, Beijing, 100084, China

[3] Key Laboratory for Thermal Science and Power Engineering of Ministry of Education, Beijing Key Laboratory of CO_2 Utilization and Reduction Technology, Department of Energy and Power Engineering, Tsinghua University, Beijing 100084, China

[4] State Key Laboratory of Integrated Optoelectronics, College of Electronic Science and Engineering, Jilin University, 2699 Qianjin Street, Changchun 130012, China

*Corresponding author. Email: linlh2019@mail.tsinghua.edu.cn (L.L.);

zcli@tsinghua.edu.cn (Z.L.);

hbsun@tsinghua.edu.cn (H.-B.S.)

Supplementary Figures

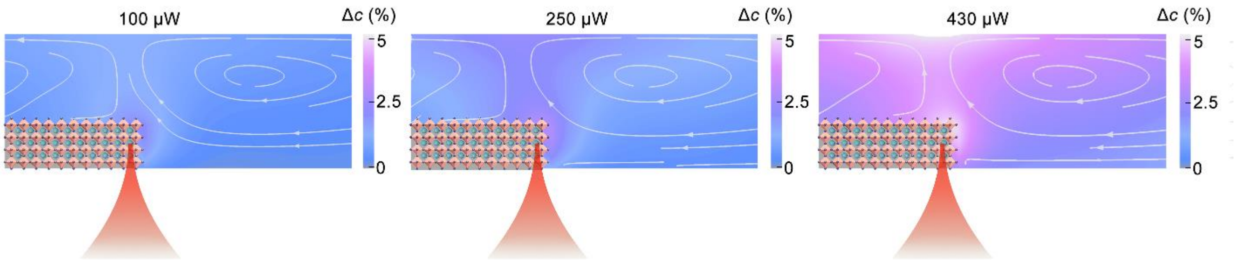

Supplementary Fig. 1. The simulation results of the supersaturation at different laser power. The left, middle, and right panel corresponds to the laser power of 100 μW, 250 μW, and 430 μW, respectively. The supersaturation can be tuned by laser power.

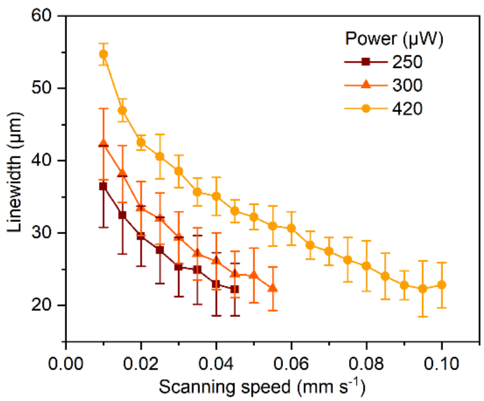

Supplementary Fig. 2. The relationship between linewidth, scanning speed of spot (i.e., growth speed) and laser power. The error bars show the standard deviation. n = 15 independent replicates. Source data are provided as a Source Data file.

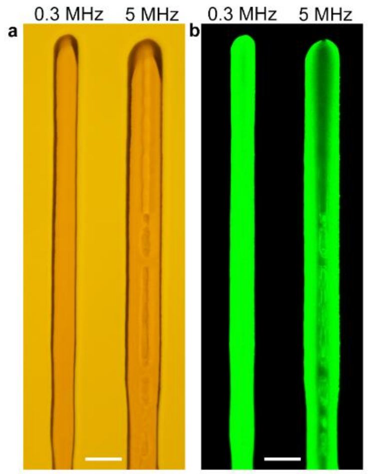

Supplementary Fig. 3. **Suppressed laser damage by reducing the repetition rate of fs-laser.** **a,** Optical images of the structures fabricated by fs-laser with repetition frequencies of 0.3 MHz (left) and 5 MHz (right), respectively. **b,** Confocal fluorescence images of the structures fabricated by fs-laser with repetition frequencies of 0.3 MHz (left) and 5 MHz (right), respectively. At the same optical intensity of 2.5×10^4 W cm^{-2}, femtosecond laser with high repetition frequency causes crystal damage, although the peak power is lower. Scale bar: 20 µm.

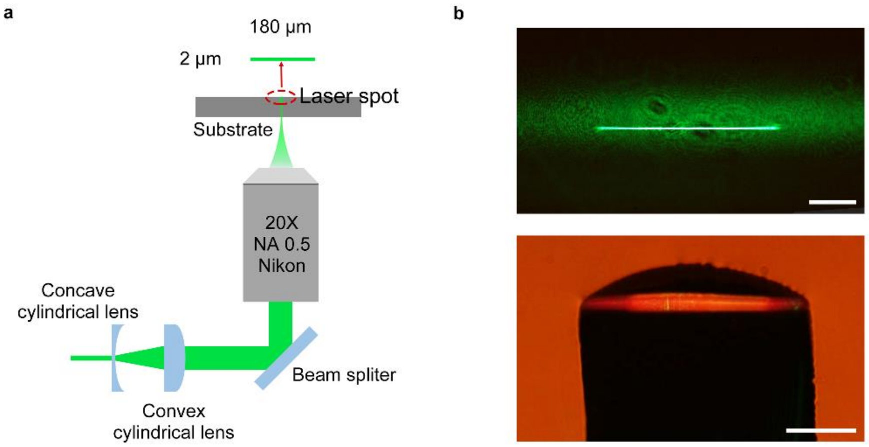

Supplementary Fig. 4. Optical printing of halide perovskites using a line-shaped laser beam. a, Schematic of the optical setup. A set of concave and convex cylindrical lenses were used to create a line pattern of 2 µm in width and 180 µm in length (after a 20 × objective). **b**, The optical images of the line-shaped laser beam (top) and the $MAPbI_3$ thin film created (bottom). A heater was used to maintain the processing temperature at 80 °C. Scale bar: 50 µm.

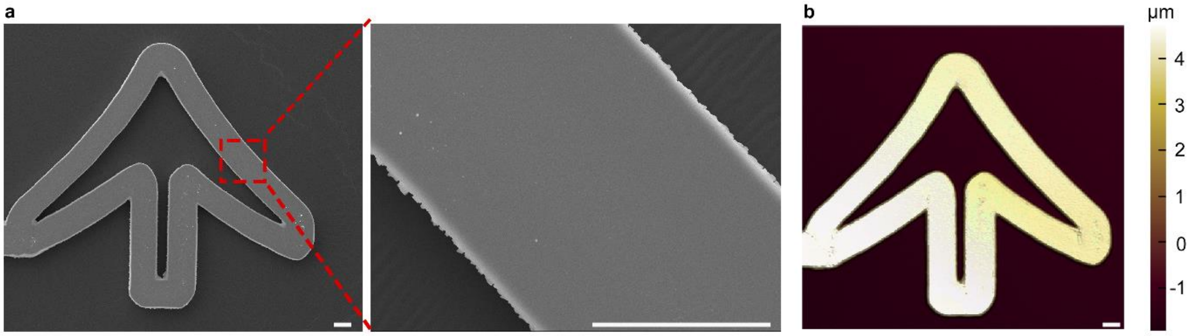

Supplementary Fig. 5. SEM and white light interferometry characterization of a grown $MAPbBr_3$ arrow pattern. a, Overall and high-magnification SEM images of the pattern. Scale bar: 10 μm. **b**, White light interferometry image of the pattern. The calculated roughness is R_q = 6.58 nm. Scale bar: 10 μm.

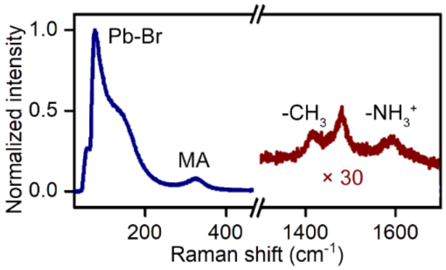

Supplementary Fig. 6. Raman characterization of printed $MAPbBr_3$ structure. The Raman peaks lower than 200 cm^{-1} arise from symmetric and asymmetric stretching modes of Pb-Br bonds, while the peak at 323 cm^{-1} corresponds to the torsion mode of MA group. The three peaks at 1427, 1478, and 1588 cm^{-1} are assigned as the symmetric bending mode of $-CH_3$, symmetric bending mode of $-NH_3^+$, and asymmetric bending mode of $-NH_3^+$, respectively.[1] Source data are provided as a Source Data file.

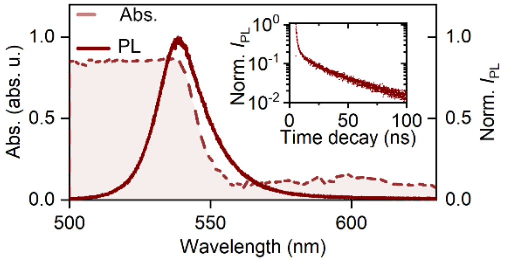

Supplementary Fig. 7. Absorption spectrum, photoluminescence spectrum and time-resolved photoluminescence measurement of $MAPbBr_3$. Source data are provided as a Source Data file.

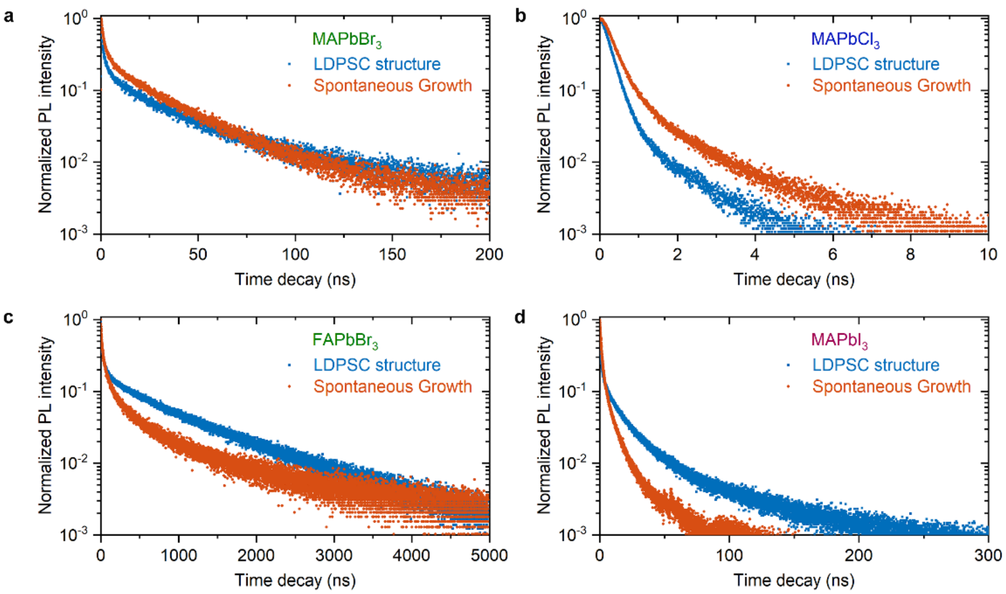

Supplementary Fig. 8. Time-resolved photoluminescence measurement of a, $MAPbBr_3$, **b,** $MAPbCl_3$, **c,** $FAPbBr_3$ and **d,** $MAPbI_3$. The laser-direct-printing-of-single-crystal (LDPSC) structure is comparable to the spontaneous growth structure, suggesting that the laser-induced defects are negligible. Source data are provided as a Source Data file.

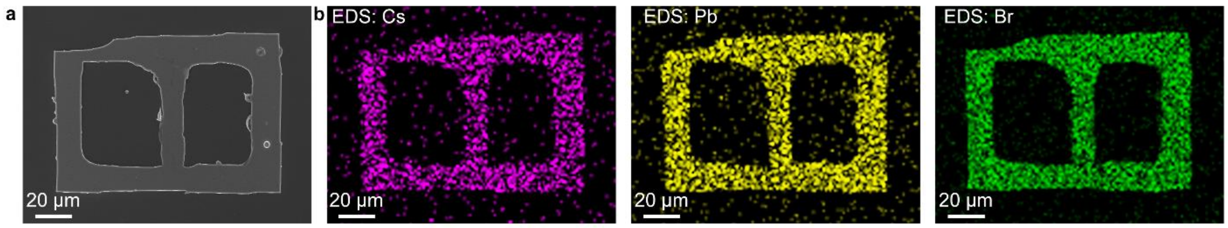

Supplementary Fig. 9. Scanning electron microscopy (SEM) and energy-dispersive X-ray spectroscopy images of $CsPbBr_3$ structure. a, SEM images of the $CsPbBr_3$ structure. **b,** The corresponding EDS mapping results. The left, middle, and right panel corresponds to the EDS results of Cs, Pb, and Br, respectively. The precursor is prepared by mixing 0.202 g CsBr and 0.697 g $PbBr_2$ in 1 mL DMSO, then adding 1 µL octylamine in 80 µL solution. The structure has a flat surface and an even distribution of elements. Scale bar: 20 µm.

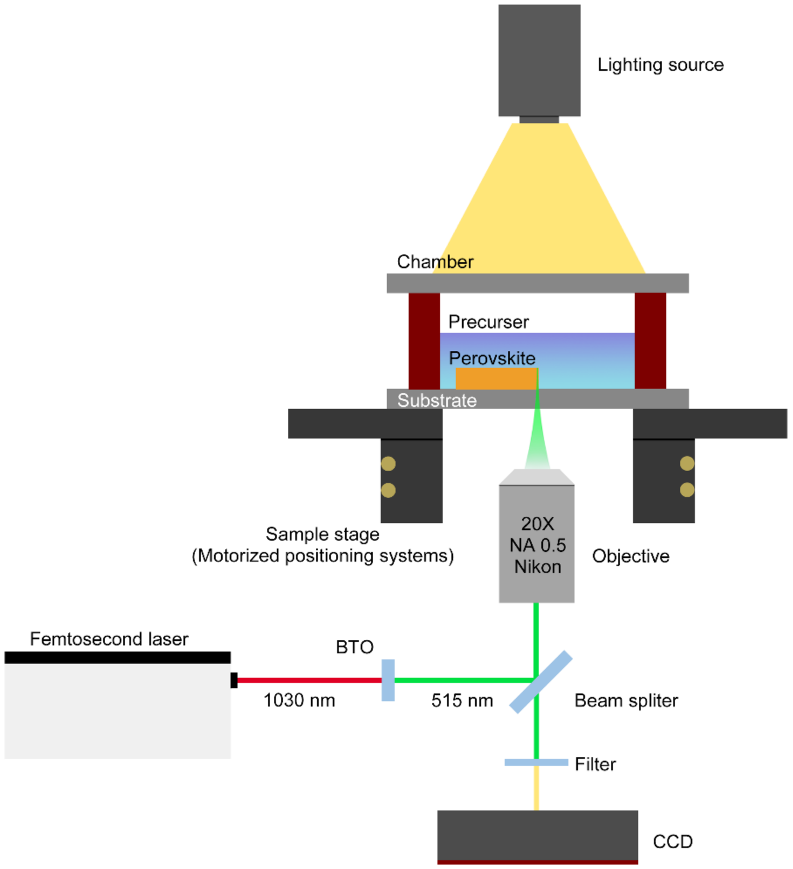

Supplementary Fig. 10. Setup for OCL fabrication.

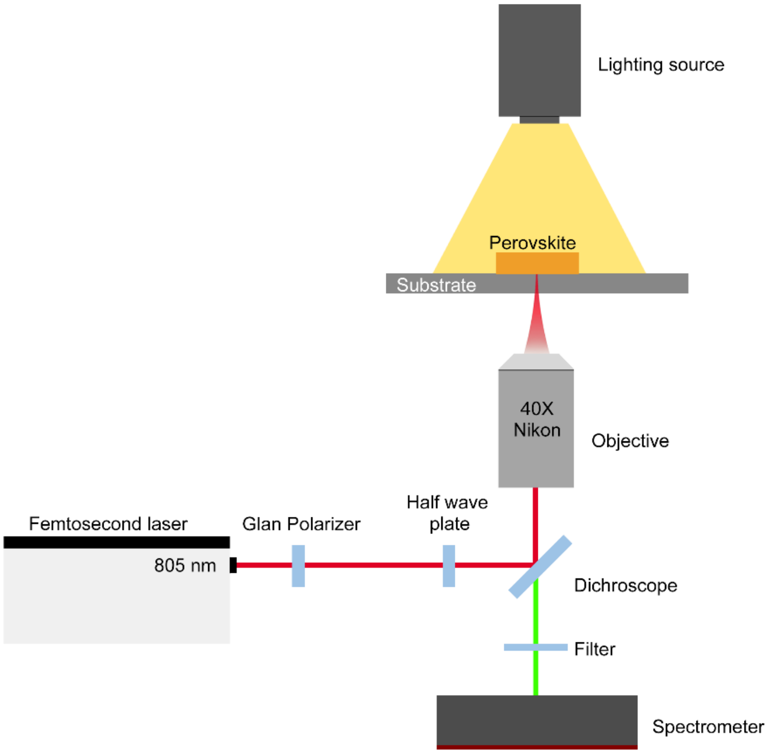

Supplementary Fig. 11. Setup of the crystal orientation characterization by two-photon fluorescence.

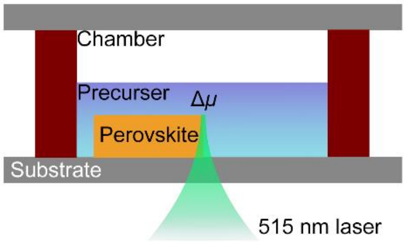

Supplementary Fig. 12. Schematic of OCL technique. $\Delta\mu$ means the laser-induced local supersaturation.

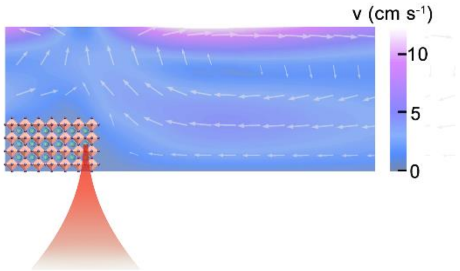

Supplementary Fig. 13. Simulation results of Marangoni convective flow.

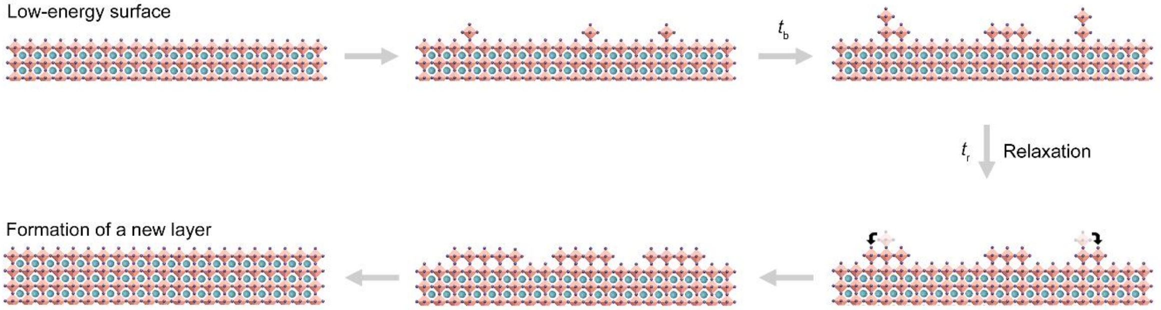

Supplementary Fig. 14. The schematic of spontaneous growth mode of perovskite. The growth begins with the bonding of atoms in solution to the surface. Although the subsequent solute atoms can bind with interface randomly, the following relaxing process will rearrange the positions of the atoms under the control of Gibbs free energy, which makes the new atoms preferentially form a flat layer. The interface grows layer by layer to expand to the direction perpendicular to the interface.

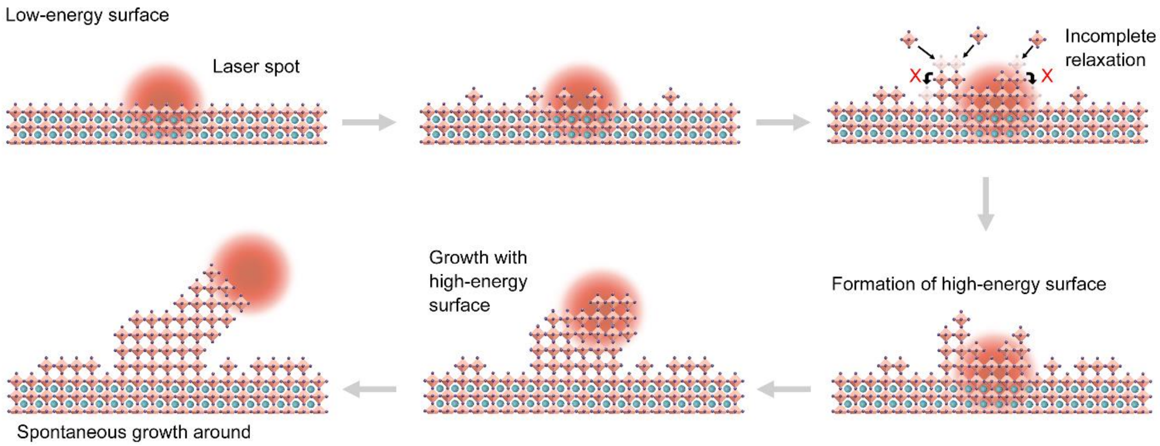

Supplementary Fig. 15. The schematic of the laser-direct-printing growth mode. When laser power is high enough, the supersaturation is also large so that the number of atoms bonding to perovskite surface per unit time increased significantly. These atoms cannot relax to the lowest energy position efficiently ($t_b < t_r$). As a result, these atoms cannot maintain a low-energy surface as they grow. At this time, the supersaturation is high and the growing interface has higher energy, so the growth rate is significantly improved compared with spontaneous growth. Eventually perovskite grows along the laser spot and exposes a high-energy surface.

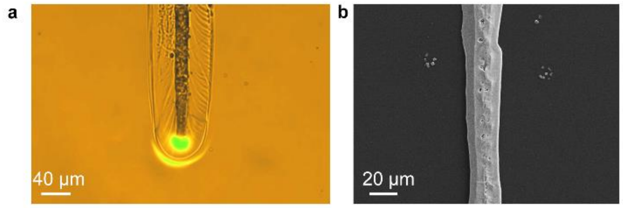

Supplementary Fig. 16. Damage in the middle of perovskite induced by continuous wave (CW) laser. a, Optical image of CW laser-induced perovskite growth. Scale bar: 40 µm. **b,** SEM image of perovskite structure fabricated by OCL with CW laser. The punctate defects in the central area are obvious. Scale bar:20 µm. Laser power: 300 µW.

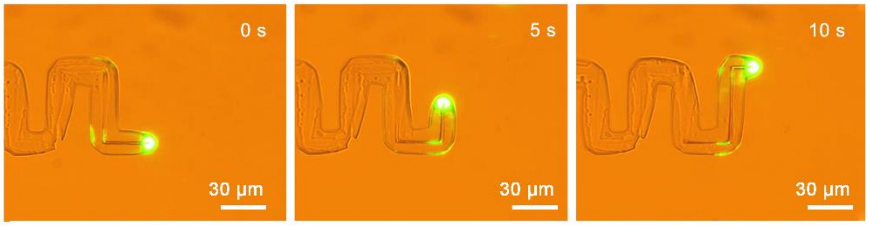

Supplementary Fig. 17. Snapshots of $MAPbBr_3$ structure growing in DMSO precursor. The left, middle, and right panel corresponds to the optical image at 0 s, 5 s, and 10 s, respectively. Scale bar: 30 µm.

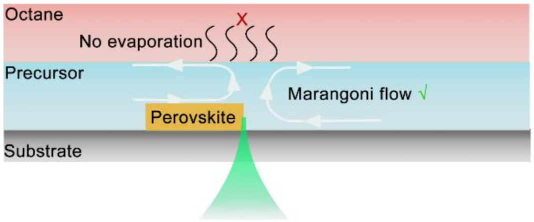

Supplementary Fig. 18. Setup to prevent evaporation and maintain convection flow. The precursor is sealed by octane. Strong Marangoni convective flow can be observed because of the existence of interface, but perovskite does not grow.

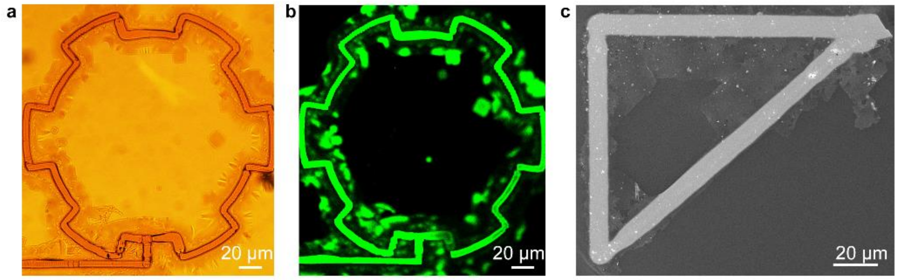

Supplementary Fig. 19. Printed halide perovskite structures with only oleylamine (OAm) as ligand. a, Optical image of a “gear” pattern. **b,** Confocal image of the “gear” pattern. The thin film surrounding the structure is also fluorescent. **c,** SEM image of a triangle pattern. The thin perovskite film surrounding the structure should be noted. Scale bar: 20 µm.

Supplementary Tables

Supplementary Table 1. Classification of Raman peaks of $MAPbCl_3$, $FAPbBr_3$ and $MAPbI_3$.

$MAPbCl_3$		$FAPbBr_3$		$MAPbI_3$	
Raman peaks (cm^{-1})	Vibration mode	Raman peaks	Vibration mode	Raman peaks	Vibration mode
100 - 183	Modes of Cl-Pb-Cl.[1]	50 - 200	Modes of Br-Pb-Br.[2]	65 - 200	Modes of I-Pb-I. [1]
488	Torsion mode of MA.[3]	309	Out-of-plane bending mode of HN–C–NH.[4]	249	Torsion mode of MA.[3]
1249	Rocking mode of MA.[5]	522	H_2N–C–NH_2 bending mode.[4]	960	Stretching mode of C-N.[6]
1483	Symmetric bending mode of NH_3^+.[5]	1391	In-plane bending mode of C–H.[2]		
1592	Asymmetric bending mode of NH_3^+.[5]	1560	Bending mode of C-H.[2]		
		1624	Stretching mode of NH_2.[2]		

Supplementary Notes

Supplementary Note 1. The working principle of OCL

We select methylammonium tribromolead ($MAPbBr_3$) as an example to illustrate the concept of OCL. In this system, laser irradiates on halide perovskite microcrystals to form a temperature gradient at liquid-gas interface and finally triggers local supersaturation (Supplementary Fig. 12).

1.1 Optothermal conversion

OCL originates from the photon absorption of the perovskites. Halide perovskite materials have high absorption coefficient for the photons whose energy is higher than the band gap. In our case, the absorption coefficient α of $MAPbBr_3$ for 515 nm laser is ≈ 5×10^{-3} nm^{-1}.[7] Most of the laser can be absorbed by perovskite. Part of the energy is dissipated through non-radiative channels and converted into heat. Besides, the low thermal conductivity of $MAPbBr_3$ and DMF (0.51 W m^{-1} K^{-1} and 0.1816 W m^{-1} K^{-1}), respectively) is conducive to the formation of local thermal field.[8,9] This temperature field has been characterized by simulations and experiments in main text.

1.2 Laser-induced local supersaturation

The construction of local temperature field will further produce local supersaturation. First, the increase of temperature accelerates the interfacial evaporation, which is the key factor to create the supersaturation environment. The evaporation rate can be calculated from the Hertz-Knudsen equation:

$$J = \frac{p}{\sqrt{2\pi m k_B T}} \tag{1}$$

In this equation, J is the flux that molecules evaporate from solvent to air, m is the molecular weight, k_B is the Boltzmann's constant, T is the temperature of the surface, and p is the corresponding saturated vapor pressure.

Usually, the evaporation rate is improved at higher temperature because p is more sensitive to temperature T, which can be described by Wagner equation:

$$\ln\left(\frac{p}{p_c}\right) = \left(\mathrm{A}_1\tau + \mathrm{A}_2\tau^{1.25} + \mathrm{A}_3\tau^3 + \mathrm{A}_4\tau^7\right)\frac{T_c}{T} \quad (2)$$

Where $\tau = 1\text{-}T/T_c$, T_c is the critical temperature and p_c is the critical pressure. A_n (n=1,2,3,4) is the coefficients which can be determined by experiments. For DMF, T_c = 596.6 K and p_c = 5.220 MPa. A_n can be obtained in Ref.[10] The calculated J was used for further COMSOL simulation. The reduction of solute molecules due to evaporation at the interface leads to supersaturation at the laser spot.

The thickness of the liquid film is about 10 micrometers, which is significantly larger than that of the spin coated films and allows the formation of thermal convective flow. Under the local temperature gradient, the surface tension difference at the interface forms a strong Marangoni convection (Supplementary Fig. 13), which influences the solute distribution in the precursor solution.[11] In addition, the temperature field can also lead to the local non-isothermal diffusion process, affecting the concentration distribution of the precursor. These factors were comprehensively considered by COMSOL simulation, which shows that a local, stable and controllable supersaturation distribution is generated around the spot (Supplementary Fig. 1). When the laser power is increased, the local solute concentration (or supersaturation) is improved as it accelerates the evaporation, the convective flow, and the thermal diffusion. The

power dependence of OCL allows us to adjust the supersaturation and perovskite growth by changing the laser power.

Supplementary Note 2. Optical power threshold in laser direct printing based on OCL

It is noted that both nucleation and crystal growth may occur when local supersaturation is created. However, crystal growth occurs more easily than nucleation because the latter has to overcome an additional energy barrier. Thus, there is no new nuclei appear around the laser spot.

When the laser power is lower than the threshold P_{th}, spontaneous growth of the perovskite crystal is observed (known as spontaneous growth mode). Macroscopically, the surface of perovskite microplates maintains the crystal orientation of low-energy during crystal growth to minimize the Gibbs free energy (Supplementary Movie 1). Microscopically, the crystal growth occurs by bonding atoms in solution to the solid-state surface. The average time for perovskite atoms to be bonded on the surface is defined as t_{b}. Although the atomic bonding is random, the following relaxation process rearranges the atomic location to minimize the Gibbs free energy. The average time of relaxation process is defined as t_{r}. Since $t_{\mathrm{b}} > t_{\mathrm{r}}$, the relaxation process always happens efficiently and makes the new atoms preferentially form a flat layer. After a new surface is filled, new atoms will be bonded on the new surface to realize crystal growth perpendicular to the surface (Supplementary Fig. 14).

When the laser power is above the P_{th}, the supersaturation is large enough so that t_{b} become shorter. When $t_{\mathrm{b}} < t_{\mathrm{r}}$, there are many randomly bound solute atoms on the perovskite surface because the atoms cannot relax efficiently and maintain low-energy surface (Supplementary Fig. 15). On the one hand, the supersaturation is high, and the atoms in the solution are more likely to overcome the potential barrier and bond to the surface. On the other hand, because of the random

arrangement of atoms on the surface, the surface energy is significantly improved. Thus, the growth rate is significantly increased compared with that of spontaneous growth mode. In other words, the growth mode is switched from spontaneous growth mode to laser-direct-printing mode. In this situation, perovskite grows rapidly and orientation of the crystal surface is defined by the laser scanning pathway (Supplementary Movie 2).

Supplementary Note 3. Avoiding the laser-induced damage on perovskite

Although OCL can drive crystal growth of perovskite, perovskite structures can also be damaged by laser due to its instability. In our experiments, we found that CW laser can produce obvious defects in the center of printed perovskite structure (Supplementary Fig. 16). When we used a femtosecond laser and reduced the frequency, these defects will gradually weaken and disappear. Similar phenomena also exist in $FAPbBr_3$ and other perovskites. This may be due to the decrease of the frequency which attenuates the thermal effect of the laser.

Supplementary Note 4. Excluding optical force as the driven force

A focused beam of light exerts a force on colloidal particles, driving them to the center of spot, which is known as optical force.[12] Optical force is a possible mechanism for the growth of perovskite. It is necessary to exclude optical force as the driven force of crystal growth. If the optical force is the main factor of crystal growth, the laser with different wavelength will have similar effect when the power is comparative. We found that when we used 660 nm CW laser which will not be absorbed by $MAPbBr_3$, the perovskite crystal did not change significantly even at the power of 0.5 W. Therefore, optical force is not the driven force of the crystal growth.

Supplementary Note 5. Excluding temperature dependence of solubility as the driving force

The solubility of $MAPbBr_3$ in DMF will decrease when the temperature is growing.[13] The solubility difference can even reach 20% from room temperature to 318 K, which can drive strong supersaturation. So, it is necessary to discuss the contribution of solubility change. We tried $MAPbBr_3$/DMSO precursor in which the solubility will increase as the temperature is growing. If the supersaturation of crystal growth is mainly provided by temperature dependence of solubility, growth will not happen in $MAPbBr_3$/DMSO precursor. However, we found that laser-induced growth can also be achieved in $MAPbBr_3$/DMSO precursor, which excludes temperature dependence of solubility as the driving force (Supplementary Fig. 17). Besides, when there is no gas-liquid interface, growth will not happen. These two experiments demonstrate that the growth of perovskite is not due to the temperature dependence of solubility.

Supplementary Note 6. Distinguishing the effects of evaporation and flow in OCL

The growth process always accompanies with evaporation and strong Marangoni flow triggered by the temperature gradient at the gas-liquid interface. It is necessary to distinguish the roles of evaporation and Marangoni flow in OCL. Octane was used to distinguish their effects. Octane will float on the precursor to seal the precursor and hinder evaporation, while the interface between octane and precursor permits Marangoni flow (Supplementary Fig. 18). The experiment showed strong Marangoni convection, while perovskite did not grow, which indicates that evaporation is the source of local supersaturation. It is noted that local and controllable supersaturation for perovskite growth can be obtained only by the synergistic effect of evaporation and convection.

Supplementary Note 7. Selection of surface ligands

The surface ligand should be carefully selected according to the composition of halide perovskites. For $MAPbBr_3$, $MAPbCl_3$ and $MAPbI_3$, oleic acid (OA) and OAm were selected as ligand, which are commonly used in the fabrication of perovskite quantum dots. OAm alone can suppress the spontaneous growth of perovskite. However, a thin-film structure appears spontaneously and affect the origin structure (Supplementary Fig. 19). The addition of OA and OAm can prevent spontaneous growth while avoiding the thin-film structure, but OA alone cannot suppress the spontaneous growth.

For $FAPbBr_3$ and $CsPbBr_3$, octylamine was selected as ligands, which is another commonly used ligand in quantum dots. OA/OAm cannot form large and uniform structure.

Supplementary references

1. Niemann, R. G. *et al.* Halogen effects on ordering and bonding of $CH_3NH_3^+$ in $CH_3NH_3PbX_3$ (X = Cl, Br, I) hybrid perovskites: A vibrational spectroscopic study. *J. Phys. Chem. C* **120**, 2509-2519 (2016).
2. Ibaceta-Jaña, J. *et al.* Vibrational dynamics in lead halide hybrid perovskites investigated by Raman spectroscopy. *Phys. Chem. Chem. Phys.* **22**, 5604-5614 (2020).
3. Maalej, A. *et al.* Phase transitions and crystal dynamics in the cubic perovskite $CH_3NH_3PbCl_3$. *Solid State Commun.* **103**, 279-284 (1997).
4. Wang, L., Wang, K. & Zou, B. Pressure-induced structural and optical properties of organometal halide perovskite-based formamidinium lead bromide. *J. Phys. Chem. Lett.* **7**, 2556-2562 (2016).
5. Glaser, T. *et al.* Infrared spectroscopic study of vibrational modes in methylammonium lead halide perovskites. *J. Phys. Chem. Lett.* **6**, 2913-2918 (2015).
6. Théorêt, A. & Sandorfy, C. The infrared spectra of solid methylammonium halides—II. *Spectrochim. Acta* **23**, 519-542 (1967).
7. Leguy, A. M. *et al.* Experimental and theoretical optical properties of methylammonium lead halide perovskites. *Nanoscale* **8**, 6317-6327 (2016).
8. Haeger, T., Heiderhoff, R. & Riedl, T. Thermal properties of metal-halide perovskites. *J. Mater. Chem. C* **8**, 14289-14311 (2020).
9. Cai, G., Zong, H., Yu, Q. & Lin, R. Thermal conductivity of alcohols with acetonitrile and N,N-Dimethylformamide. *J. Chem. Eng. Data* **38**, 332-335 (1993).
10. Cui, X., Chen, G. & Han, X. Experimental vapor pressure data and a vapor pressure equation for N,N-dimethylformamide. *J. Chen. Eng. Data* **51**, 1860-1861 (2006).
11. Sun, J. K., Sobolev, Y. I., Zhang, W., Zhuang, Q. & Grzybowski, B. A. Enhancing crystal growth using polyelectrolyte solutions and shear flow. *Nature* **579**, 73-79 (2020).
12. Grier, D. G. A revolution in optical manipulation. *Nature* **424**, 810-816 (2003).
13. Saidaminov, M. I., Abdelhady, A. L., Maculan, G. & Bakr, O. M. Retrograde solubility of formamidinium and methylammonium lead halide perovskites enabling rapid single crystal growth. *Chem. Commun.(Cambridge, U.K.)* **51**, 17658-17661 (2015).
