# Supplementary material for: Optofluidic crystallithography for directed growth of single-crystalline halide perovskites
Source: Nat Commun. 2024 May 1;15:3677. doi: 10.1038/s41467-024-48110-w (PMC11063063; doi:10.1038/s41467-024-48110-w)
Supplement: Supplementary file 3 — Description of Additional Supplementary Files [file 41467_2024_48110_MOESM3_ESM.pdf]

Description of Additional Supplementary Files

File Name: Supplementary Movie 1
Description: Improved spontaneous growth of $MAPbBr_3$ at a low optical power.

File Name: Supplementary Movie 2
Description: The printing of $MAPbBr_3$ micro gear structure using optofluidic crystallithography (OCL).

File Name: Supplementary Movie 3
Description: The printing of $MAPbBr_3$ at different ligand concentrations.
